# Expression ratio of circular to linear ANRIL in hypertensive patients with coronary artery disease

**DOI:** 10.1038/s41598-022-05731-9

**Published:** 2022-02-02

**Authors:** Iman Razeghian-Jahromi, Mohammad Javad Zibaeenezhad, Ali Karimi Akhormeh, Mahintaj Dara

**Affiliations:** 1 Cardiovascular Research Center, Shiraz University of Medical Sciences, Shiraz, Iran; 2 Stem Cell and Transgenic Technology Research Center, Shiraz University of Medical Sciences, Shiraz, Iran

**Keywords:** RNA, Cardiovascular diseases, Epigenetics, Gene expression, Gene regulation, Genetic markers, RNA splicing

## Abstract

Atherosclerotic lesions of the coronary arteries are still in charge of significant annual morbidity and mortality despite intense therapeutic advancements. Genome-born elements contribute substantially to the atherosclerosis process. ANRIL is one of the long non-coding RNAs with outstanding functions particularly regulation of genes involved in atherosclerosis development. In this study, we measured ANRIL expression (circular-, linear-, and circular/linear ratio) in hypertensive patients with coronary artery disease (CAD) compared with peers without CAD. Among hypertensive patients who were candidates of angiography, 25 subjects with CAD and the equal number without CAD were considered as the case and control groups, respectively. Different categories of data were recorded through a predefined questionnaire. Before angiography, blood samples were obtained. After RNA extraction and cDNA synthesis, quantitative PCR was performed using specific primers for circular and linear ANRIL. Age and gender were not different between the groups. Most of the parameters of the lipid profile besides creatinine and blood urea nitrogen were remarkably worse in the case group. Circular ANRIL was significantly lower in the case group while linear counterparts were significantly higher in this group. Circular/linear ratio was also significantly lower in the case group. To overcome growing devastating trend of CAD, scrutinizing different factors involved in the initiation and development of atherosclerosis is a must. Atheroprotective role of circular ANRIL and atheroprogressive role of linear ANRIL were shown in our patients with hypertension.

## Introduction

The greatest share in cardiovascular toll has been allocated to coronary artery disease (CAD)^1^. Genetic traits contribute substantially to the development of atherosclerosis in the coronary arteries^2^. About 98% of the transcribed RNAs in the cell have no open reading frames, i.e. no capacity to produce protein^3^. Earlier, they were considered as transcriptional noise. However, harboring a wealth of information revealed their potential for regulation of different mechanisms. One type of these untranslated elements is long non-coding RNAs (lncRNAs) with more than 200 nucleotides long^4^. They modulate genes' expression in order to fine-tune biological processes like cell growth and development, angiogenesis, and inflammation^5–8^. Accordingly, footprints of lncRNAs are evident in many disorders such as cancers, Alzheimer disease, and cardiovascular impairments^4,9,10^.

Genome wide association studies demonstrated that atherosclerotic vascular disease, CAD, stroke, myocardial infarction, and aortic aneurysm are all associated with chromosome 9p21^11–15^. This type of association is independent of traditional risk factors like hypertension, obesity, smoking, or lipid profile disorders. The INK4/ARF locus at chromosome 9p21 contains the codes for three tumor suppressor genes. Transcription of this gene cluster in the antisense direction produces a 3.8 kb lncRNA named Antisense Non-coding RNA in the INK4 Locus (ANRIL, also known as CDKN2B-AS or CDKN2B-AS1)^16,17^. ANRIL is implicated in different components of the atherosclerosis process like vascular endothelial cells, vascular smooth muscle cells, mononuclear phagocytes, and atherosclerotic plaques^18–20^. Regulation of gene expression is the prime function of ANRIL^21^. Other than locally, ANRIL also works remotely via chromatin modulation, binding to transcription factors, and regulation of miRNAs^22^. Furthermore, ANRIL is underwent complex tissue-specific pattern of splicing which leads to the production of multiple isoforms^23,24^. Also, Circular ANRIL, discovered in 2012, is a derivative of linear ANRIL that is subjected to back-splicing^25,26^. Based on its linearity or circularity abundance, it may have promoting or protecting role for atherosclerosis. Moreover, ANRIL could be a potent diagnostic and prognostic indicator for coronary heart disease (CHD)^21^. Elucidation of differential gene expression is useful for understanding the pathogenesis of atherosclerosis, which in turn smooths the road for more efficient preventive, and therapeutic interventions^21^.

The importance of hypertension is not only related to the fact that it is among the most strong driving factors for atherosclerotic plaque formation^27^, but it also augments the adverse effects of other cardiovascular risk factors in CAD patients^28^. Lack of association between Chr9p21 locus and hypertension indicates that ANRIL possibly exert its function through a novel mechanism^29^. On the other hand, an association between ANRIL levels and hypertension was reported^30^. In one study, both ANRIL and hypertension were shown to be independent risk factors for restenosis in CAD patients, and interaction between ANRIL and hypertension was statistically significant^31^. As it seems, there is a complicated relation between hypertension and ANRIL expression. In order to investigate the role of ANRIL in atherosclerosis exclusively, the study was conducted on hypertensive patients (diastolic blood pressure of ≥ 90 mmHg and/or systolic blood pressure of ≥ 140 mmHg^32^).

The expression of ANRIL in diabetic CAD patients was previously investigated^30^. Our hypothesis is that there might be a difference in the expression level of circular and linear ANRIL as well as circular/linear ratio in hypertensive patients with CAD compared with non-CAD peers.

## Materials and methods

This study was in accordance with the Declaration of Helsinki, and approved by the regional ethics committee (Shiraz University of Medical Sciences) by ID "IR.SUMS.REC.1397.990". Patients who were suspicious to CAD, and referred for angiography were considered during DEC-2019 to FEB-2021. Decision for doing angiography was made by an interventional cardiologist according to the American College of Cardiology guideline^33^. In catheterization laboratory, only patients with hypertension were included. Hypertension was defined as systolic and diastolic blood pressure of equal or more than 140/90 mmHg^32^. Those with previous experiences of heart diseases (except for coronary atherosclerosis), chronic kidney disease, obstructive pulmonary disorders, and any other inflammation were excluded. Case group (n = 25) were hypertensive patients with CAD and control group (n = 25) were hypertensive patients without CAD, according to the angiography findings.

A predefined questionnaire was used by a nurse to obtain participant's data. Some of these data like age, gender, fasting blood sugar (FBS), total cholesterol, high density lipoprotein-cholesterol (HDL-C), low density lipoprotein-cholesterol LDL-C, triglycerides (TG), TG/HDL-C ratio, LDL/HDL ratio, creatinine, and blood urea nitrogen (BUN) were extracted from blood analysis report. Some of the others like smoking status, education stage, and existence of dyslipidemia, diabetes, and history of coronary atherosclerosis besides family history for hypertension, diabetes, atherosclerosis, and sudden death were asked from the patients themselves. Other ones like systolic and diastolic blood pressure, overweight, and body mass index (BMI) were measured by the nurse.

Before angiography, blood samples were collected in anticoagulant tubes, and stored at – 80 °C until use. The expression analysis of circular and linear ANRIL was carried out through reverse transcriptase polymerase chain reaction (RT-PCR) assay. After full thawing at room temperature, samples were subjected to RNA extraction by RNX-plus kit (Cinagen, Iran) in accordance to the manufacturer instructions. Afterwards, cDNA was synthesized with SMO-BIO cDNA synthesis Kit (Taiwan). cDNA was then used as a template for downstream quantitative PCR with specific primers. GAPDH was used for internal control. Primer sequences were as follows:

**Table:** 

	Forward primer (5′–> 3′)	Reverse primer (5′–> 3′)
Circular ANRIL (162 bp)	GAATTTTGACAGTGTCCCTTTTG	CTCTCTTTCCAAGAAAATTCTCC
Linear ANRIL (208 bp)	GCAGCTTTCTGCTACATGGAG	CTATATGCTTGGGCAAATCAC
GAPDH (110 bp)	TGACAACGAATTTGGCTACAGC	CTCTTCCTCTTGTGCTCTTGC

### Statistical analysis

According to the sample size, normality of variables was checked by Shapiro–Wilk test. Continuous variables were expressed as mean ± SD, and categorical variables were presented as number (percent). Independent sample t test, Mann–Whitney test, and chi-square-test were used to compare continuous and categorical variables, as appropriate. The SPSS 26 for Windows was used for statistical analysis (SPSS, Inc., Chicago, IL, USA). For gene expression analysis, 2^−ΔΔCt^ method was applied to determine fold changes expression of genes. Normality was checked by kurtosis and skewness indices as well as Q–Q plot. We used independent sample t test for analyzing Ct data.

### Ethical statement

This study was in accordance to the Declaration of Helsinki, and approved by the regional ethics committee, IR.SUMS.REC.1397.990.

### Consent to participate

Written informed consent was obtained from each patient to take part in the study.

### Consent for publication

The authors declare that the personal information of each patient would be confidential. The patients had taken their consent for publication of data before enrolling in the study.

## Results

The mean age of the patients was 57.3 ± 11.6 years with dominance of female gender. Both groups were hypertensive with no significant difference neither in systolic, nor in diastolic blood pressure. FBS and BMI were not different between the two groups. Unlike triglycerides, lipid profile including total cholesterol, HDL-C, and LDL-C were significantly undesirable in the case group. Moreover, TG/HDL-C ratio and LDL/HDL ratio as well as creatinine and BUN were significantly higher in the case group (Table 1).

**Table 1:** Comparison of continuous variables between control and case groups.

	Total (n = 50)	Control (n = 25)	Case (n = 25)	*P*	Reference range
Age	57.3 ± 11.6	53.8 ± 9.6	60.9 ± 12.6	0.085	–
Systolic blood pressure (mmHg)	149.5 ± 26.6	157 ± 29	142 ± 22	0.052	Less than 120 mmHg
Diastolic blood pressure (mmHg)	82.7 ± 11.7	81.4 ± 9.8	84 ± 13.3	0.254	Less than 80 mmHg
FBS (mg/dl)	120 ± 51	129 ± 56	111 ± 44	0.696	70 -99 mg/dL
Total Cholesterol (mg/dl)	173 ± 38	158 ± 32	188 ± 37	**0.003**	Less than 200 mg/dL
HDL-C (mg/dl)	43.1 ± 8.1	48.1 ± 6.1	37.9 ± 6.06	** < 0.001**	Optimal: equal or more than 60 mg/dL
LDL-C (mg/dl)	92.8 ± 35.4	73.6 ± 24.8	112.9 ± 33.9	** < 0.001**	Less than 100 mg/dL
TG (mg/dl)	187 ± 53	191 ± 59	181 ± 46	0.872	Less than 150 mg/dL
TG/HDL-C ratio	4.47 ± 1.43	4.04 ± 1.33	4.91 ± 1.41	**0.022**	Ideal: 2.0 or less high: 4.0—6.0
LDL/HDL ratio	2.3 ± 1.18	1.54 ± 0.051	3.1 ± 1.16	** < 0.001**	Ideal: below 2.0 good: below 5.0
Cr (mg/dl)	1.01 ± 0.23	0.92 ± 0.24	1.1 ± 0.18	**0.002**	For adult men, 0.74 to 1.35 mg/dL For adult women, 0.59 to 1.04 mg/dL
BUN (mg/dl)	17.1 ± 6.3	14.7 ± 4.7	19.5 ± 6.9	**0.005**	7–20 mg/dl
BMI (kg/m^2^)	27.6 ± 3.7	28 ± 3.4	27.2 ± 4	0.393	18.5 – 24.9 kg/m^2^

*FBS* fasting blood sugar, *HDL-C* high density lipoprotein-cholesterol, *LDL-C* low density lipoprotein-cholesterol, *TG* triglycerides, *Cr* creatinine, *BUN* blood urea nitrogen, *BMI* body mass index. Data are presented as mean ± SD. Bold values imply statistical significance (P < 0.05).

Smoking habit and history of coronary atherosclerosis were significantly evident in the case group while gender, overweight, diabetes and dyslipidemia were almost similar between the two groups. It may be interesting to know that illiteracy was remarkably higher in patients with CAD (Table 2).

**Table 2:** Comparison of categorical variables between control and case groups.

	Total (n = 50)	Control (n = 25)	Case (n = 25)	Odds ratio (CI)	*P*
Gender (male)	20 (40%)	7 (28%)	13 (52%)	2.8 (0.86–9.01)	0.087
Smoking (n = 48)	19 (39.6%)	6 (25%)	13 (54.2%)	3.54 (1.04–12.06)	**0.043**
Education (illiterate)	27 (54%)	9 (36%)	18 (72%)	4.57 (1.38–15.11)	**0.013**
Overweight	38 (76%)	21 (84%)	17 (68%)	0.4 (0.1–1.6)	0.192
Dyslipidemia (n = 49)	28 (57.1%)	11 (44%)	17 (70.8%)	3.1 (0.95–10.08)	0.053
Diabetes	19 (38%)	9 (36%)	10 (40%)	1.2 (0.38–3.72)	0.771
History of coronary atherosclerosis (n = 49)	11 (22.4%)	0	11 (44%)	13.6 (1.26–2.5)	** < 0.001**

*CI* confidence interval: Data are presented in numbers (%). Bold values imply statistical significance (P < 0.05).

We also sought family history of hypertension, diabetes, atherosclerosis, and sudden death. In contrary to atherosclerosis and diabetes, family history of hypertension and sudden death were significantly obvious in the control group (Table 3).

**Table 3:** Comparison of family history between control and case groups.

	Total (n = 50)	Control (n = 25)	Case (n = 25)	P (Fisher’s exact test)
Hypertension	23 (46%)	17 (68%)	6 (24%)	**0.002**
Diabetes	12 (24%)	8 (32%)	4 (16%)	0.160
Atherosclerosis	20 (40%)	12 (48%)	8 (32%)	0.193
Sudden death	12 (24%)	10 (40%)	2 (8%)	**0.009**

Data are presented in numbers (%). Bold values imply statistical significance (P<0.05).

Table 4 depicted univariate and multivariate analyses of ANRIL expression levels in the control and case groups. While expression level of circular ANRIL was significantly lower in the case group, linear ANRIL was expressed significantly higher in this group. Furthermore, we calculated the expression ratio of circular/linear ANRIL in the both groups. Case group show significantly lower value in comparison to the control groups. Furthermore, multivariate analyses with incorporation of different patterns of cardiovascular risk factors were demonstrated in Table 4. In the case of including maximum number of risk factors, only linear ANRIL expression remained statistically different in the case versus control group.

**Table 4:** Univariate and multivariate analyses of ANRIL expression between control and case group.

	Control	Case	Unadjusted *P*	P*	P**	P***
Circular ANRIL	1.00 ± 0.43	0.76 ± 0.37	**0.042**	0.081	**0.043**	0.157
Linear ANRIL	1.00 ± 0.64	2.86 ± 1.65	** < 0.0001**	**0.001**	**0.015**	**0.043**
Circular/Linear ANRIL	1.00 ± 0.51	0.39 ± 044	** < 0.0001**	**0.003**	**0.010**	0.053

Bold values imply statistical significance.

*Adjusted for age, sex, and BMI.

**Adjusted for age, sex, BMI, diabetes, dyslipidemia, and hypertension.

***Adjusted for age, sex, BMI, diabetes, dyslipidemia, hypertension, smoking Habit, and history of atherosclerosis.

The association between severity of atherosclerosis and ANRIL expression was shown in Table 5. There was no statistical difference between single-, two-, or three-vessel disease and circular-, linear-, or circular/linear ANRIL expression.

**Table 5:** The association between atherosclerosis severity and ANRIL expression.

	SVD	2VD	3VD	*P*
Circular ANRIL	0.849 ± 0.387	0.848 ± 0.347	0.711 ± 0.397	0.700
Linear ANRIL	2.982 ± 2.059	3.068 ± 1.577	2.763 ± 1.686	0.931
Circular/linear ANRIL	0.615 ± 0.831	0.240 ± 0.066	0.384 ± 0.400	0.474

*SVD* single vessel disease, *2VD* two vessel disease, *3VD* three vessel disease.

## Discussion

In the present study, circular-, linear-, and circular/linear ANRIL expression were compared between hypertensive patients with CAD and peers without CAD. Our findings demonstrated that the expression of circular ANRIL was lower in the CAD patients while linear ANRIL was higher in this group. This possibly proposes an atheroprotection role for circular ANRIL, and an atheroprogressive role for linear ANRIL. Furthermore, difference of linear ANRIL in the case over the control group is more statistically significant than that of the circular isoform between the two groups. This means that linear ANRIL is in a stronger association with atherosclerotic lesions than circular ones. Notably, significant difference in the expression ratio of circular to linear ANRIL in the case compared with the control group could be a valuable indicator for diagnosis of CAD. While lipid profile impairments were obvious in the case group, family history of hypertension and sudden death were unexpectedly more in the control peers. It necessitates further studies to test any relationship between changes in ANRIL and abnormal lipid profile. All in all, given that atherosclerosis is a multifactorial disorder, its progression is influenced by a combination of different determinants. So, assuming a causal association between ANRIL expression and emergence of atherosclerosis should be inspected in future prospective investigations.

ANRIL, as the main gene in the chromosome 9p21 CAD locus, has been attracted a great attention in the recent years^22,34^. Intriguingly, there is no homolog for ANRIL transcript in mice, and it seems that ANRIL-related functions are specific to the humans^35^. Proliferation, migration, senescence, and apoptosis of vascular smooth muscle cells, which all are in close association with atherosclerosis process are influenced by ANRIL expression^35–38^. In each cell type, ANRIL transcription is specifically regulated, and is followed by certain modulations in the cellular processes. Indeed, another level of regulation is performed via splicing that leads to variation in abundance of ANRIL in different cell types^22^. In turn, some cellular processes like genotoxic stress, tumorigenesis, senescence, and inflammation alter ANRIL expression^22^.

Generation of circular RNAs is somewhat competitive with that of linear ones^39^. In the ANRIL context, the pattern of splicing may change from sequential order to back-splicing to form circular variant during some conditions like stress. This means that circular RNA is not necessarily a functional transcript, and it could be a byproduct of alternative splicing. Compelling evidence show that the expression level of several hundreds of circular RNAs are even 10 folds higher than their linear ones^26,40^. Circular RNAs are more stable and useful for regulation of homeostasis^41^.

Atheroprotection that is originated from 9p21 locus is associated with low linear and high circular ANRIL expression. It seems that the level of expression of the two isoform types in relation with each other is a critical factor for determination of atherosclerosis development. In other interesting words, circularization could be considered as a genius strategy that deployed by the cell to prevent adverse effects of linear ANRIL (onset and progression of atherosclerosis)^42,43^ which directs vascular tissues to a more healthy status. However, single nucleotide polymorphisms (SNPs) in the 9p21 locus are also in charge of differential expression of ANRIL isoforms between subjects. Indeed, the expression level of circular ANRIL is suspected to a mosaicism in primary smooth muscle cells and macrophages of the vascular tissues^44^. So, there is a need to scrutinize the role of circular ANRIL in decreasing the risk of atherogenesis at single-cell scale.

CAD is considered as an inflammatory and proliferative disorder^44,45^. The pathogenesis of CAD is explained with the involvement of a plethora of chemokines, cytokines, and growth factors, which act combinatorial in different stages^46^. Inflammatory diseases including CAD change the expression of lncRNAs. This unique feature render lncRNAs as diagnostic biomarkers and therapeutic targets^47,48^. To substantiate, ANRIL level was found to be associated with IL6/IL8 levels in blood which indicates a relationship between ANRIL and CAD^30^.

Age, diabetes status, and hypertension are among the factors that change the levels of ANRIL^30^. However, a study concluded that ANRIL expression is not correlated with the lipid profile^49^. In our study, there were no differences in some cardiovascular risk factors like age, gender, FBS, hypertension, TG, BMI, and diabetes between CAD positive and negative participants. In consistent with other study, higher level of linear ANRIL translates into increased atherosclerosis^50^. Similar to our finding, the expression of circular and linear ANRIL was reported to be inversely correlated^51^. Those patients with overexpressed circular ANRIL and highest circular/liner ANRIL ratio develop less CAD. Expression of linear ANRIL was not changed by circular ANRIL overexpression^44^. In another investigation, five lncRNAs were measured in the peripheral blood of patients with myocardial infarction (MI) who underwent percutaneous coronary intervention. ANRIL was reported to be upregulated in this population^30^.

ANRIL was upregulated in diabetic patients with CHD (2.34 folds) compared with peers without CHD^52^. ANRIL expression was significantly increased in a population of diabetic patients with CAD possibly due to imposed dysregulation in neighboring or inflammatory genes. After adjusting for confounding variables, only linear ANRIL was significantly different between CAD and non-CAD patients. This shows superior value of linear ANRIL in atherosclerosis studies compared with circular- and circular/linear ratio.

However, we did not find any association between ANRIL expression, even linear isoform, and atherosclerosis severity (single vessel- versus multivessel disease) in contrast to other study^50^. This discrepancy may be originated from differences in the genetic or environmental factors of the studied population which affect the development and severity of atherosclerosis. In one study on patients with acute MI, four lncRNAs including ANRIL were differentially expressed. Also, all the lncRNAs were associated with hypertension and left ventricular dysfunction, one of the lncRNA was found to be in relation with diabetes, and the association of the other one with smoking was confirmed^30^.

Inconsistent findings about ANRIL are entirely possible. For instance, one SNP increases ANRIL expression followed by pro-proliferative function in the endothelial, macrophage, or vascular smooth muscle cells resulting in atherosclerosis while another polymorphism at the same position decreases proliferative isoforms in the beta cells promoting diabetes^22^. The underlying reasons for such disparities among the studies may be due to the splicing complexity of ANRIL and differences in the sample-originated factors like ethnicity, age, and health status^35^. Splicing variants and binding sites in the cells and tissues are among the sophisticated features of ANRIL that remain to be elusive^21^.

In therapeutic view, circular ANRIL could be a potent tool for treatment of atherosclerosis and other proliferative disorders based on its stability against degradation and antiatherogenic-antiproliferative characteristics^44^. Although therapeutic activation of circular ANRIL seems to be beneficial for inhibiting atherosclerosis progression, it will be accompanied by certain side effects since ANRIL have multiple targets other than cardiovascular system. These challenges limit the studies on ANRIL^21^.

## Limitations

It would be better if we had ANRIL expression levels in normotensive subjects with and without CAD. A more comprehensive assessment of ANRIL expression in relation to high blood pressure and CAD pathogenesis was feasible in this way. Some data, as previously mentioned in the methods section, were asked from the patients themselves that may be in contrast to the documented findings from blood analysis report. In this case, the parameters of blood analysis is more reliable. Also, defining certain cutoff values of ANRIL expression corresponding to different CAD severity merits future investigation.

## Conclusion

Circular-, linear-, and circular/linear ANRIL were differentially expressed in hypertensive CAD patients compared with those without CAD.
